# Editorial: Microbe-nanoparticle interactions: a mechanistic approach

**DOI:** 10.3389/fmicb.2023.1273364

**Published:** 2023-08-18

**Authors:** Madhuree Kumari, Sylvia Natalie Klodzinska, Mariana Carmen Chifiriuc

**Affiliations:** ^1^Department of Biochemistry, Indian Institute of Science, Bengaluru, India; ^2^Department of Pharmacy, University of Copenhagen, Copenhagen, Denmark; ^3^Department of Botanic and Microbiology, University of Bucharest, Bucharest, Romania

**Keywords:** nanoparticles, microbes, interactions, drug delivery, biofilm, resistance

The last decade has seen a tremendous growth in nanoparticles' application as potential antimicrobials against human, plant and animal diseases. The recent developments in synthesis and use of metallic, magnetic, polymeric nanoparticles, liposomes, nanoemulsions and nanogels have paved a path for their successful application against drug resistant bacteria, fungi and viruses. The nanoparticles mediated drug-delivery systems and their crucial role against quorum sensing and biofilms have also been studied at a large scale.

Microbes including bacteria, fungi and viruses undergo a series of biochemical, biophysical, physiological, molecular and metabolic changes during their interaction with different nanosystems. Nanoparticles employ a series of cellular and epigenetic changes including generation of oxidative stress, DNA damage, changes in subcellular structure, change in omics to degrade the microbial structure and serving as a potential antimicrobial agent. However, some reports of microbes resistant against metal nanoparticles have also been reported. The current Research Topic aimed to address the mechanistic insights into the interaction of microbes-nanoparticles as potential antimicrobials and the strategies in use to prevent the development of microbial resistant against nanoparticles systems. Within this Research Topic, five articles including three research articles, one review and one mini-review were published, which added to our knowledge on microbe-nanoparticle interaction.

The research article of Dhanya Raj et al. focused on the increasing drug resistance patterns in uropathogens and role of emerging nano-therapeutics. They synthesized silver nanoparticles using a brown-seaweed *Turbinaria ornate*. The obtained particles were spherical in nature, with an average size of 73.98 nm. The synthesized particles showed a high antioxidative and antibacterial activities. A good antibacterial activity was observed against uropathogens, including *Staphylococcus aureus, Escherichia coli, Pseudomonas aeruginosa, Enterococcus faecalis*, and *Klebsiella pneumoniae*. The authors concluded that silver nanoparticles can be developed as a new class of antimicrobial agents against urinary tract infections.

Tiwari et al. worked on the development of “surface-tuned biocompatible nanomaterial-containing formulations with selective antimicrobial activity.” They focused on understanding the mechanism of polyethyleneimine-functionalized silver nanoparticles (PEI-f-Ag-NPs) as an antifungal agent using the intrinsic fluorescence of whole *Candida albicans* cells as a molecular probe. PEI-f-Ag-NPs showed a low MIC value of 5 μg/mL, with a rapid killing kinetics. Silver nanoparticles had a strong binding tendency with *C. albicans* surface and had a more selective interaction with the tyrosine-rich proteins in the fungal cell. The study concluded that PEI-f-AG-NPs were able to generate reactive oxygen species and form cell wall pit while acting as antifungal agent.

Further investigating the nanoparticle-microbe interaction, Ameh et al. reported the role of nanoparticles surface stabilizing agents in determining their antibacterial action. Cetyltrimethylammonium bromide (CTAB, to confer a positive surface charge) and polyvinyl pyrrolidone (PVP, to confer a neutral surface charge) were used as surface stabilizing agents for the synthesis of silver and copper nanoparticles. The results indicated that CTAB stabilized silver and copper nanoparticles were more effective antibacterial agents against *E. coli, S. aureus* and *Sphingobacterium multivorum* than PVP stabilized metal nanoparticles. In conclusion, the authors specified the important of the surface charge and stabilizing agents of nanoparticles in conferring the antibacterial potential to them.

Furthermore, a review by Modi et al. shed light on nanoparticle surface-bacterial membrane interactions in overcoming antibiotic resistance. They also focused on the role of surface functionalization of nanoparticles with aptamers and antibodies. Nanocarriers can employ a number of mechanisms including interaction with bacterial cell wall, lipopolysaccharide, cell membrane, ROS mediated bacterial killing, and targeting ion channels and efflux proteins to kill the bacterial cells. The authors concluded on designing smart surface-functionalized nanocarriers which can act as bacteria- targeted robots with the potential to replace the conventional antibiotics.

Last but not the least, Kamat and Kumari in their mini-review presented the latest development in nanoparticle-microbe interaction, “emergence of microbial resistance against nanoparticles.” They reviewed an important concern of nanoparticle resistance acquired by some bacterial pathogens. They summarized the mechanisms employed by pathogens to gain resistance against nanoparticles: nanoparticle transformation-induced oxidative stress, membrane alterations, reversible adaptive resistance, irreversible modifications to cell division, and a change in bacterial motility and resistance. Furthermore, they suggested the important strategies which can be employed to prevent the pathogens from acquiring resistance against the nanoparticles.

In conclusion, the Research Topic was presented with multiple interesting and scientific articles previding the insights on nanoparticle-microbe interaction. The editors consider that this issue has been successful in reporting on recent studies on the mechanistic of nanoparticle-microbe interaction.

## Author contributions

MK: Conceptualization, Project administration, Resources, Supervision, Writing—original draft. SK: Conceptualization, Project administration, Supervision, Writing—review and editing. MC: Project administration, Supervision, Writing—review and editing.

